# Host Susceptibility to *Brucella abortus* Infection Is More Pronounced in IFN-****γ**** knockout than IL-12/****β****2-Microglobulin Double-Deficient Mice

**DOI:** 10.1155/2012/589494

**Published:** 2011-12-11

**Authors:** Ana Paula M. S. Brandão, Fernanda S. Oliveira, Natalia B. Carvalho, Leda Q. Vieira, Vasco Azevedo, Gilson C. Macedo, Sergio C. Oliveira

**Affiliations:** ^1^Department of Biochemistry and Immunology, Institute of Biological Sciences, Federal University of Minas Gerais, 31270-901 Belo Horizonte, MG, Brazil; ^2^Department of General Biology, Institute of Biological Sciences, Federal University of Minas Gerais, 31270-901 Belo Horizonte, MG, Brazil; ^3^Department of Parasitology, Microbiology and Immunology, Biological Sciences Institute, Federal University of Juiz de Fora, 36036-900 Juiz de Fora, MG, Brazil

## Abstract

*Brucella abortus* is a facultative intracellular bacterial pathogen that causes abortion in domestic animals and undulant fever in humans. IFN-**γ**, IL-12, and CD8+ T lymphocytes are important components of host immune responses against *B. abortus*. Herein, IFN-**γ** and IL-12/**β**2-microglobulin (**β**2-m) knockout mice were used to determine whether CD8+ T cells and IL-12-dependent IFN-**γ** deficiency would be more critical to control *B. abortus* infection compared to the lack of endogenous IFN-**γ**. At 1 week after infection, IFN-**γ** KO and IL-12/**β**2-m KO mice showed increased numbers of bacterial load in spleens; however, at 3 weeks postinfection (p.i.), only IFN-**γ** KO succumbed to *Brucella*. All IFN-**γ** KO had died at 16 days p.i. whereas death within the IL-12/**β**2-m KO group was delayed and occurred at 32 days until 47 days postinfection. Susceptibility of IL-12/**β**2-m KO animals to *Brucella* was associated to undetectable levels of IFN-**γ** in mouse splenocytes and inability of these cells to lyse *Brucella*-infected macrophages. However, the lack of endogenous IFN-**γ** was found to be more important to control brucellosis than CD8+ T cells and IL-12-dependent IFN-**γ** deficiencies.

## 1. Introduction

*Brucella* is a Gram-negative bacterium which is pathogenic to humans and animals [1]. The establishment on infection depends of entrance of this bacterium through the nasal, oral, and/or conjunctival mucosa. After entering into the host cells, *Brucella* has the ability to infect and multiply in phagocytic and nonphagocytic cells [2, 3]. However, macrophages are considered the main cells of *Brucella* residence in the host [4]. The immune response against *Brucella* infection involves many molecules and cells to trigger a Th1 immune response and activation of CD8+ T cells [5–7].

IFN-*γ* is a critical cytokine for host control of *Brucella* infection [8–10]. The importance of IFN-*γ* to control *Brucella* was first shown in vivo with monoclonal antibodies that depleted or neutralized IFN-*γ* in mice [10–12]. Subsequently, a more dramatic role was shown by using IFN-*γ* KO mice when both BALB/c and C57BL/6 mice died after infection with *B. abortus* strain S2308 [8]. CD4+ T cells are the major producers of IFN-*γ* in brucellosis, although other subsets such as CD8+ T cells also contribute [7, 13]. A number of studies have demonstrated a role for either CD4+ or CD8+ T cells in the control of brucellosis [7, 14]. In adoptive transfer studies, CD8+ and CD4+ T cells have been shown to be equally protective for resistance to infection with virulent *B. abortus *[15]. Using *β*2-microglobulin (*β*2-m) gene KO mice, our group has demonstrated that CD8+ T cells have an additional role of lysing infected macrophages and thus either killing intracellular *Brucella* or exposing them to IFN-*γ*-activated macrophages [7].

IL-12 is a proinflammatory cytokine that has a profound effect on the induction of IFN-*γ*-producing type 1 pattern of immune response during *Brucella* infection [9, 16]. Since CD8+ T cells and IL-12 are important immunological components during brucellosis, we decided to investigate the course of *Brucella* infection in IL-12/*β*2-microglobulin double KO mice compared to IFN-*γ* KO animals. This study was designed to determine whether CD8+ T cells and IL-12-dependent IFN-*γ* deficiencies would be more critical to control *B. abortus* infection compared to the lack of endogenous IFN-*γ*. Our results revealed that IFN-*γ* and IL-12/*β*2-m KO mice died from *Brucella* infection. However, all IFN-*γ* KO were dead at day 16 postinfection (p.i.) whereas death within the IL-12/*β*2-m KO group was delayed and occurred at day 32 until day 47. These results suggest that lack of endogenous IFN-*γ* is more important than CD8+ T cells and IL-12-dependent IFN-*γ* deficiencies to control murine brucellosis.

## 2. Materials and Methods

### 2.1. Mice

IL-12/*β*2-microglobulin double-deficient mice (IL-12/*β*2-m^−/−^) were provided by Dr. Leda Quercia Vieira (UFMG, Belo Horizonte, Brazil), and IFN-*γ*, deficient mice (IFN-*γ* ^−/−^) were provided by Dr. João Santana Silva (USP, Ribeirão Preto-SP, Brazil). The wild-type strain C57BL/6 mice were purchased from the Federal University of Minas Gerais (UFMG, Belo Horizonte, Brazil). Genetically deficient and control mice were maintained at UFMG and used at 6–8 weeks of age.

### 2.2. Bacteria

*Brucella abortus* S2308 strain was obtained from our laboratory collection [17]. The strain S2308 was grown in Brucella Broth liquid medium (BB) (DIFCO) at 37°C under constant agitation (200 rpm). After three days of growth, the bacterial culture was centrifuged and the pellet was resuspended in saline (NaCl 0.8% wt/vol), divided in aliquots, and frozen in 20% glycerol (vol/vol). Aliquots of these cultures were serially diluted and plated on BB medium containing 1.5% bacteriological agar (wt/vol). After incubation for 72 hours at 37°C, bacterial numbers were determined by counting colony forming units (CFU).

### 2.3. Infection and *Brucella* Counting in Spleens

Five mice of each strain (IL-12/*β*2-m^−/−^, IFN-*γ* ^−/−^, or C57BL/6) were infected intraperitoneally with 1 × 10^6^ CFU of* B. abortus* virulent strain S2308. These mice were sacrificed at 1- and 3-weeks after infection. The spleen harvested from each animal was macerated in 10 mL of saline (NaCl 0.8%, wt/vol), and it was used for counting of CFU and splenocyte culture. For CFU determination, spleen cells were serially diluted and were plated in duplicate on BB agar. After 3 days of incubation at 37°C in air with 5% CO_2_, the number of colony forming units (CFU) was determined. Results were expressed as the mean log CFU of each group. The experiment was repeated three times.

### 2.4. Measurement of Cytokines and NO into Splenocyte Culture Supernatants

Spleens cells from IL-12/*β*2-m^−/−^, IFN-*γ* ^−/−^, and C57BL/6 mice obtained after maceration were treated with ACK buffer (0.15 M NH_4_Cl, 1.0 mM KHCO_3_, 0.1 mM Na_2_EDTA, pH 7.2) to lyse red blood cells. After that, the cells were washed with saline (NaCl 0.8%, wt/vol) and suspended in RPMI 1640 (Gibco, Carlsbad, Calif) supplemented with 2 mM L-Glutamine, 25 mM HEPES, 10% (vol/vol) heat-inactivated FBS (Gibco, Carlsbad, Calif), penicillin G sodium (100 U/mL), and streptomycin sulfate (100 *μ*g/mL). To determine cytokine concentration by ELISA, 1 × 10^6^ spleen cells were plated per well in a 96-well tissue culture-treated dish. Murine splenocytes from infected animals were stimulated with *B. abortus* S2308 (MOI 100 : 1), Concanavalin A (5 *μ*g/mL Sigma, Sigma-Aldrich, St. Louis, Mo), or *E.coli* LPS (1 *μ*g/mL, Sigma, St. Louis, Mo). Unstimulated cells were used as negative control. Spleen cells were incubated at 37°C in 5% CO_2_, and aliquots of the supernatant were collected after 48 and 72 hrs of culture for TNF-*α* and IFN-*γ* measurements, respectively. Levels of TNF-*α* and IFN-*γ* were measured into cell supernatants by ELISA using the Duoset kit (R&D Systems, Minneapolis, Minn) according to the manufacturer's instructions. To assess the amount of NO produced, splenocyte culture supernatants from IFN-*γ* ^−/−^, IL-12/*β*2-m^−/−^, and C57BL/6 mice were assayed for accumulation of the stable end product of NO, NO_2_^−^ which was determined by the Griess reaction. Briefly, culture supernatants (50 *μ*L) from spleen cells stimulated as above mentioned for cytokine measurement were mixed with 50 *μ*L of Griess reagent (1% sulfanilamide, 0.1% naphthylethyline diamine dihydrochloride, and 2.5% phosphoric acid) into plates. The OD at 550 nm was then measured. NO_2_^−^ was quantified by comparison with NaNO_2_ as a standard.

### 2.5. Survival Curve

Five mice of each strain (IL-12/*β*2-m^−/−^, IFN-*γ* ^−/−^, or C57BL/6) were infected intraperitoneally with 1 × 10^6^ CFU of* B. abortus *virulent strain S2308. Percentage of mouse survival was observed during 50 days postinfection. The experiment was repeated twice.

### 2.6. Generation and *In Vitro* Stimulation of Bone Marrow-Derived Macrophages (BMDMs)

Macrophages were derived from bone marrow of IL-12/*β*2-m^−/−^, IFN-*γ* ^−/−^, and C57BL/6 mice as previously described [18]. Briefly, bone marrow (BM) cells were removed from the femurs and tibias of the animals. Each bone was flushed with 5 mL of Hank's balanced salt solution (HBSS). The resulting cell suspension was centrifuged, and the cells were resuspended in DMEM (Gibco, Carlsbad, Calif) containing 10% (vol/vol) FBS (HyClone, Logan, Utah), 1% (wt/vol) HEPES, and 10% (vol/vol) L929 cell-conditioned medium (LCCM) as source of M-CSF, in 24 well plates (5 × 10^5^ ells/well). After 4 days, 100 *μ*L/well LCCM was added. At day 7, the medium was renewed. At day 10 of culture, when the cells had completely differentiated into macrophages, the medium was harvested, and we added supplemented DMEM (500 *μ*L/well) containing *B. abortus* S2308 (MOI 1000 : 1) or *E. coli *LPS (1 *μ*g/mL, Sigma, St. Louis, Mo). Culture supernatants of BMDMs were collected after 24 hours of stimulation and assayed for the concentrations of IL-12 and TNF-*α* by ELISA (R&D Systems) according to the manufacturer's instructions.

### 2.7. Cytotoxic Assay

To determine the cytolytic activity of splenocytes from *Brucella*-infected mice, we used the CytoTox 96 Nonradioactive Cytotoxicity Assay (Promega, Madison, USA) that is based on the colorimetric detection of the released levels of the LDH enzyme. Macrophages differentiated (5 × 10^5^ cells/well) from IL-12/*β*2-m^−/−^, IFN-*γ* ^−/−^, and C57BL/6 mice were infected with *B. abortus* (MOI 100 : 1). After 24 hours of infection extracellular bacteria was removed. Macrophages infected were used as target cells for cytotoxic assay. Splenocytes (1 × 10^6^ cells/well) obtained from IL-12/*β*2-m^−/−^, IFN-*γ* ^−/−^ and C57BL/6 mice at one week p.i. were used as effector cells and were cocultured with macrophages in 24 well plates in DMEM medium. Effector cells were added to target cells in duplicate at 2 : 1 ratio. Culture was maintained at 37°C in 5% CO_2_ for 24 hours, and 50 *μ*L of supernatants were harvested and placed in 96-well flat-bottom plate. Controls for spontaneous LDH release from effector and target cells, as well as target maximum release, were also added in the experiment. The cell supernatants were assayed for lactate dehydrogenase (LDH) activity following the manufacturer's protocol. The percentage of specific lysis was calculated according to the following formula: [(Experimental−Effector Spontaneous−Target Spontaneous)/(Target Maximum−Target Spontaneous)] × 100%.

### 2.8. Statistical Analysis

The results of this study were analyzed using the Student's *t*-test, using GraphPad Prism 4 (GraphPad Software, Inc). The level of significance in the analysis was *P* < 0.05.

## 3. Results

### 3.1. Increased *B. abortus* CFU in Spleens of IFN-*γ* ^−/−^ and IL-12/*β*2-m^−/−^ Mice

**Figure 1:**
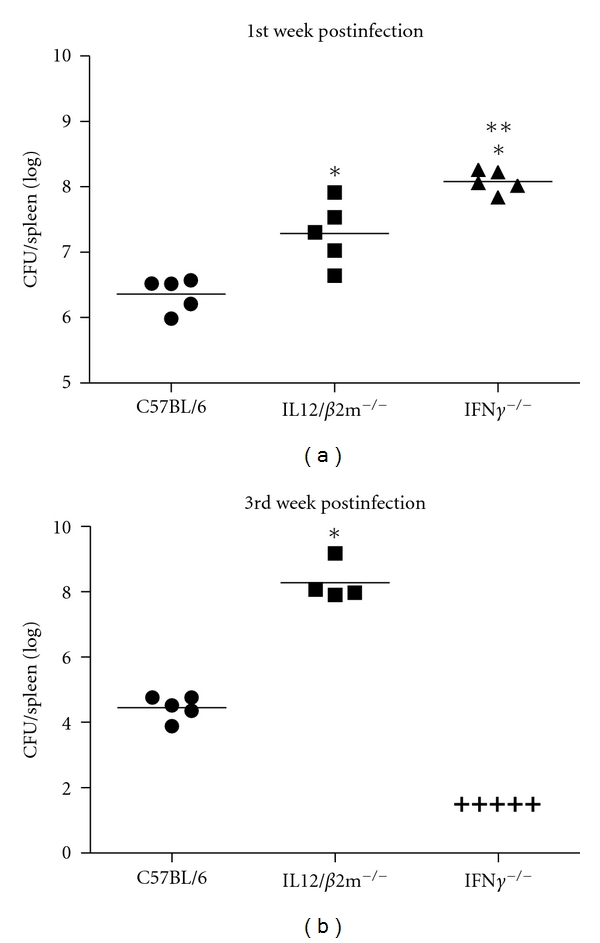
Growth of *B. abortus *in spleens of C57BL/6, IFN-*γ* ^−/−^, and IL-12/*β*2-m^−/−^ mice at the first (a) and third (b) week postinfection. IL-12/*β*2-m^−/−^, IFN-*γ* ^−/−^, and C57BL/6 mice were intraperitoneally inoculated with 10^6^ CFU of *B. abortus *S2308. (+) This symbol shows that all IFN-*γ* ^−/−^mice died before the CFU count at the third week postinfection. Data are expressed as mean ± SD of five animals per time point. These results are representative of three independent experiments. Significant difference in relation to C57BL/6 for *P *< 0.05 is denoted by an asterisk and in relation to IL-12/*β*2-m^−/−^ by two asterisks.

The level of systemic infection in murine brucellosis is detectable by enumerating the number of residual *Brucella* CFU in mouse spleens [19]. Thus, C57BL/6, IFN-*γ* ^−/−^, and IL-12/*β*2-m^−/−^ mice were infected with *B. abortus* virulent strain, and splenic CFU were counted at 1 and 3 weeks postinfection (Figure 1). At one week postinfection, IL-12/*β*2-m^−/−^ and IFN-*γ* ^−/−^ mice displayed increased numbers of *Brucella* CFU (7.28 ± 0.21 and 8.08 ± 0.07, resp.) compared to wild-type animals (6.36 ± 0.11). Additionally, the CFU difference observed between IL-12/*β*2-m^−/−^ and IFN-*γ* ^−/−^ mice was statistically significant. At 3 weeks postinfection, the difference in *Brucella* CFU from IL-12/*β*2-m^−/−^ animals compared to C57BL/6 increased from 0.92 to 3.76 logs. As for IFN-*γ* ^−/−^ mice, at 3 weeks after infection, all animals were dead. These results demonstrated enhanced susceptibility of IL-12/*β*2-m^−/−^ and IFN-*γ* ^−/−^ mice to brucellosis, being more prominent in IFN-*γ* ^−/−^ animals.

### 3.2. IFN-*γ* ^−/−^ Are More Susceptible to *B. abortus* Infection Than IL-12/*β*2-m^−/−^ Mice

**Figure 2:**
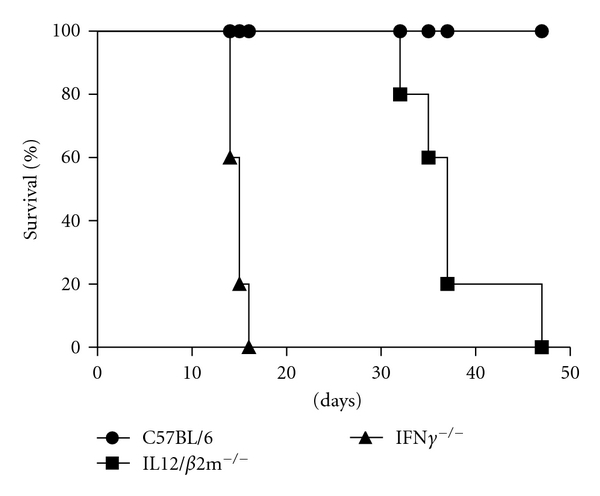
IFN-*γ* is critical for efficient control of *B. abortus *in vivo. Groups of 5 mice were injected intraperitoneally with 10^6^ CFU of *B. abortus *S2308. Each mouse strain was monitored daily for survival during 50 days postinfection.

IFN-*γ* and IL-12/*β*2-m KO on a C57BL/6 background were compared to their ability to survive *Brucella* infection. As shown in Figure 2, all IFN-*γ* KO succumbed at 16 days p.i. whereas death within the IL-12/*β*2-m KO group was delayed and occurred at 32 days until 47 days postinfection. In contrast, at 50 days p.i., 100% of C57BL/6 mice were still alive. These results suggest that IFN-*γ* and IL-12/*β*2-m are critical immune components to combat *Brucella* infection. However, the lack of endogenous IFN-*γ* is more important than CD8+ T cells and IL-12-dependent IFN-*γ* deficiencies to control murine brucellosis.

### 3.3. IFN-*γ* ^−/−^ and IL-12/*β*2-m^−/−^ Mice Showed Reduced Specific Type 1 Immune Response to *B. abortus*

**Figure 3:**
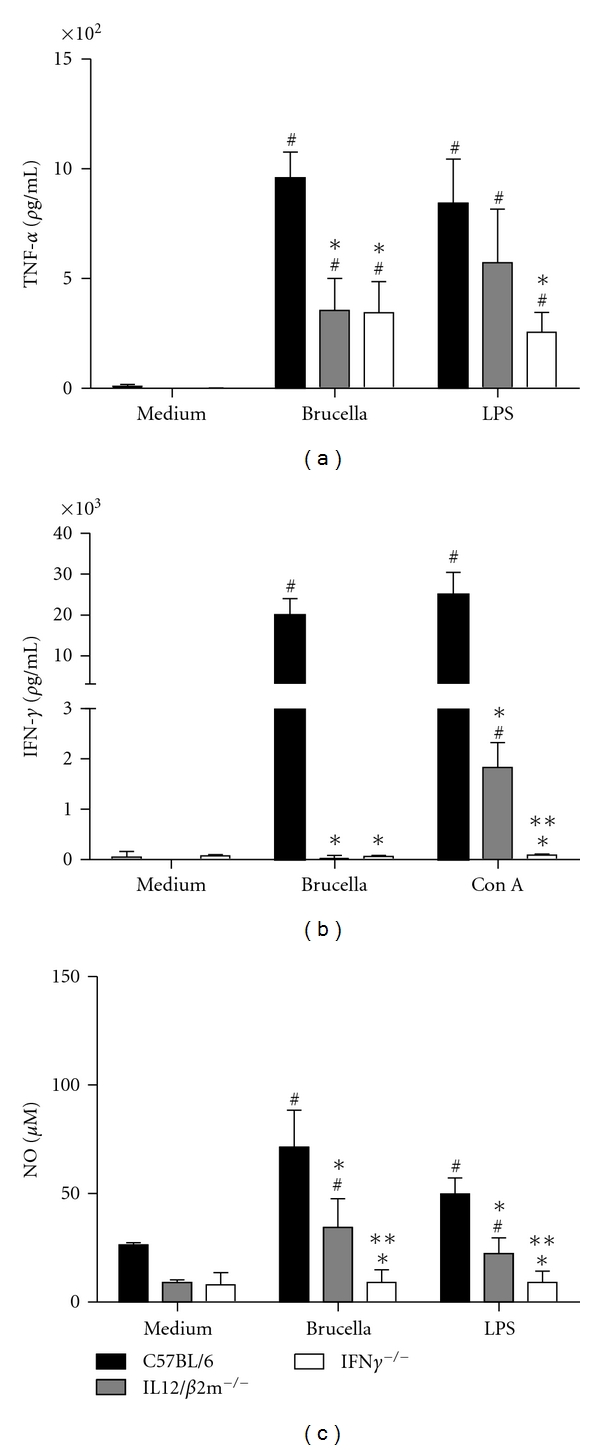
IFN-*γ*, TNF-*α*, and NO production induced by *B. abortus* in IFN-*γ* KO or IL-12/*β*2-m KO splenocytes. Spleens cells (1 × 10^6^ cells) were stimulated with *B. abortus* S2308 (MOI 100 : 1), Con A (5 *μ*g/mL), or *E*. *coli *LPS (1 *μ*g/mL). Levels of TNF-*α* (a) and IFN-*γ* (b) were measured by ELISA after 48 and 72 hrs, respectively. Levels of NO_2_^−^ (c) were measured by Griess reaction after 48 hrs of antigen stimulation. Statistically significant differences in relation to C57BL/6 mice are indicated by an asterisk (*P* < 0.05), in relation to IL-12/*β*2-m^−/−^ by two asterisks, and those between unstimulated and stimulated spleen cells are indicated by the symbol # (*P* < 0.05).

Protective immunity against infection by *B. abortus* is directly related to the induction of a type 1 pattern of immune response. IL-12 and IFN-*γ* are key cytokines involved in this type of immunity [20]. Thus, we evaluated the production of IFN-*γ*, TNF-*α*, and NO in spleen cells from IFN-*γ* ^−/−^ and IL-12/*β*2-m^−/−^ mice. As expected, no detectable IFN-*γ* production was observed in IFN-*γ* ^−/−^ and also in IL-12/*β*2-m^−/−^ mice when compared to wild-type animals at one week after infection (Figure 3(b)). Furthermore, a dramatic reduction on TNF-*α* and NO production was observed in IFN-*γ* ^−/−^ and IL-12/*β*2-m^−/−^ mice when compared to wild-type animals (Figures 3(a) and 3(c)). Additionally, the levels of NO produced by IFN-*γ* ^−/−^ cells were reduced when compared to IL-12/*β*2-m^−/−^ mice. These results demonstrate that type 1 cytokine profile is compromised in IFN-*γ* ^−/−^ and IL-12/*β*2-m^−/−^ mice during *Brucella* infection.

### 3.4. IFN-*γ* ^−/−^ and IL-12/*β*2-m^−/−^ Mice Produce Normal Levels of TNF-*α* Levels in Macrophages

**Figure 4:**
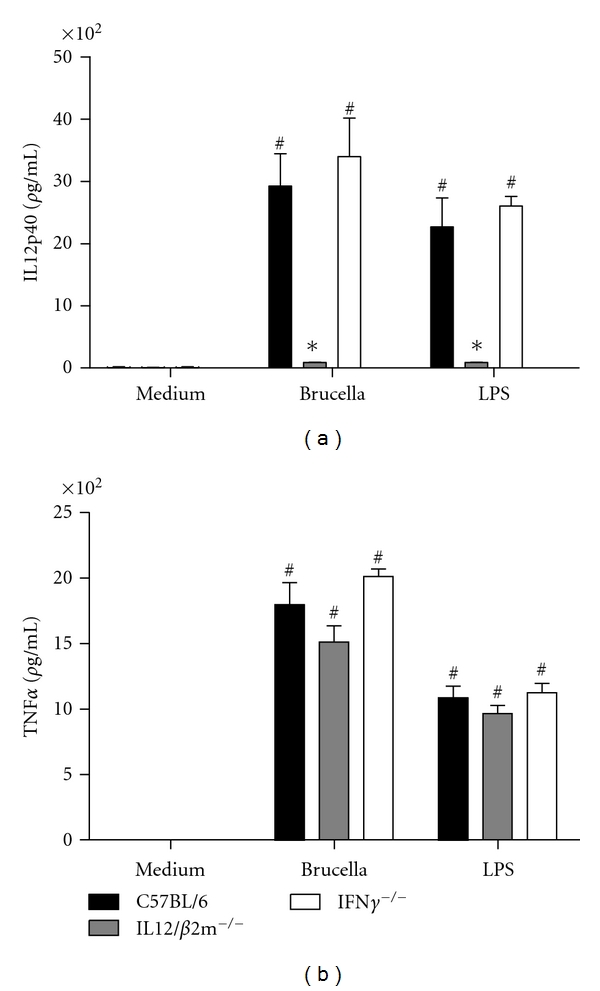
IL-12 and TNF-*α* production induced by *B. abortus* in IFN-*γ* KO or IL-12/*β*2-m KO macrophages. Bone marrow from C57BL/6, IFN-*γ* ^−/−^, and IL-12/*β*2-m^−/−^ mouse cells were differentiated in macrophages and stimulated with *B. abortus* S2308 (MOI 100 : 1) or *E. coli* LPS (1 *μ*g/mL). Supernatants were harvested for measuring IL-12 (a) and TNF-*α* (b) after 24 hrs by ELISA. Significant difference in relation to nonstimulated cells is denoted by the symbol # and an in relation to C57BL/6 mice is denoted by an asterisk (*P *< 0.05). Results are means ± standard deviations of experiments performed with three animals. Data shown are representative of two different experiments.

The recognition of *Brucella* by innate immune cells, such as macrophages and dendritic cells, results in activation and the concomitant production of proinflammatory cytokines [21]. In this study, we evaluated the proinflammatory cytokine production by macrophages from bone-marrow cells of IFN-*γ* ^−/−^ and IL-12/*β*2-m^−/−^ mice when stimulated with live *B. abortus* or *E. coli* LPS. As shown in Figure 4, no IL-12 was detected in IL-12/*β*2-m^−/−^ mice as expected but normal levels of this cytokine were measured in IFN-*γ* ^−/−^ cells. Regarding TNF-*α*, no statistically significant difference in production of this proinflammatory mediator was detected in knockout mice compared to C57BL/6.

### 3.5. Cytotoxic Activity of *B. abortus*-Induced Splenocytes

**Figure 5:**
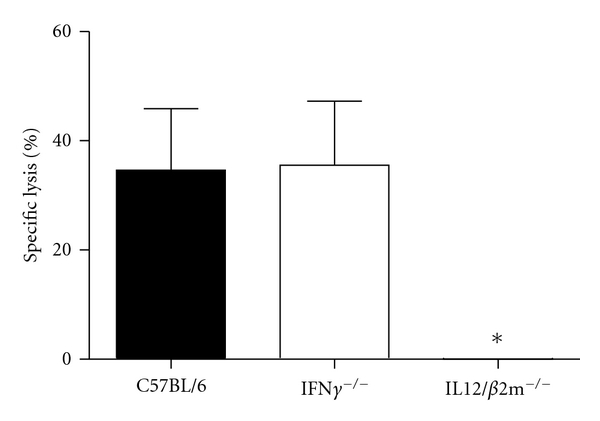
Lysis of *B. abortus*-infected macrophages by splenocytes from IFN-*γ* KO and C57BL/6 mice. Macrophages differentiated (5 × 10^5^ cells/well) obtained from IL-12/*β*2-m^−/−^, IFN-*γ* ^−/−^, and C57BL/6 mice were infected with *B. abortus* (MOI 100 : 1) and used as target cells. Splenocytes (1 × 10^6^ cells/well) obtained from IL-12/*β*2-m^−/−^, IFN-*γ* ^−/−^, and C57BL/6 mice at one week of infection were used as effector cells for cytotoxic assay and were cocultured with macrophages in 24 well plates in DMEM medium. Effector cells were added to target cells in duplicate at 2 : 1 ratio. Significant difference in relation to C57BL/6 and IFN-*γ* KO mice is denoted by an asterisk (*P *< 0.05). Results are means ± standard deviations of experiments performed. Data shown are representative of two different experiments.

The ability of *B. abortus*-primed splenocytes from IFN-*γ* ^−/−^, IL-12/*β*2-m^−/−^, and C57BL/6 mice to lyse infected bone-marrow-derived macrophages was assayed. Specific lysis of *Brucella*-infected macrophages was detected in IFN-*γ* ^−/−^ (35.5 ± 5.8) and wild-type (34.6 ± 5.6) mice but not in IL-12/*β*2-m^−/−^ animals (Figure 5). This result suggests the lack of functional CD8+ CTL in IL-12/*β*2-m^−/−^ mice what is one of the reasons for enhanced susceptibility to *B. abortus* infection in these animals.

## 4. Discussion

Typical host immunity to *Brucella* is based on a Th1-dependent immune response. Previously immunity to intracellular bacteria was considered to be exclusively dependent on CD4+ T cells [22]. However, later studies have emphasized the role of CD8+ T cells in protection against *Brucella* infection. [7, 8]. The purpose of this study was to compare the susceptibility of IFN-*γ* KO versus IL-12/*β*2-m KO animals, defining the importance of these immune components on host resistance to *B. abortus* infection.

 Previous studies have demonstrated that IFN-*γ* was indeed crucial for the control of *Brucella* infection [8, 10]. Additionally, our group and others have established that CD8+ T cells are critical components of host resistance to *Brucella* [7, 15]. Herein, we determined that IFN-*γ* KO mice had increased numbers of *Brucella* CFU compared to IL-12/*β*2-m KO at one week postinfection. Furthermore, all IFN-*γ* KO died of infection at 16 days p.i. whereas death within the IL-12/*β*2-m KO group was delayed and occurred at 32 days until 47 days postinfection. In contrast, at 50 days p.i., 100% of C57BL/6 mice were still alive. Ko et al. [23] have previously demonstrated that IL-12 KO mice infected with *B. abortus* did not control infection and maintained high plateau of bacteria; however, the animals did not die at fours week postinfection. In contrast, in our study, IL-12/*β*2-m mice succumbed to infection as a result of combined IL-12 and *β*2-m deficiencies. Taken together, these results suggest that IFN-*γ* and IL-12/*β*2-m are important components to host control of *Brucella* infection. However, the lack of endogenous IFN-*γ* is more important than CD8+ T cells and IL-12-dependent IFN-*γ* deficiencies to induce immunity to brucellosis.

 In order to determine which factors could be involved with enhanced susceptibility to *Brucella* infection in IFN-*γ* KO and IL-12/*β*2-m KO mice, we determined the concentration of IFN-*γ*, TNF-*α*, and NO in spleen cells of these animals. Splenocytes from both KO mice stimulated with live *Brucella* produced undetectable levels of IFN-*γ* and reduced amounts of TNF-*α* and NO. In the case of NO, this reduction was prominent in IFN-*γ* KO. Recently, Norman et al. have identified IFN-*γ*-based mechanisms that regulate NO production [24]. Furthermore, Yagi et al. [25] have demonstrated that deletion of Gata 3 allowed the appearance of IFN-*γ*-producing cells in the absence of IL-12. Thus, the Runx3-mediated pathway, actively suppressed by GATA3, induces IFN-*γ* production in a STAT4- and T-bet-independent manner. Another study using *Listeria monocytogenes* at low dose revealed that splenocytes of IL-12 KO mice produced only 10% of the amount of IFN-*γ* detected in wild-type mice in response to antigen [26]. They suggested that NK cells or other cells have the potential to produce residual but substantial amounts of IFN-*γ* independent of IL-12. Since *Listeria*-infected mice showed enhanced IL-18 expression, this cytokine may stimulate NK cells for IFN-*γ* production in the absence of IL-12. Additionally, Freudenberg et al. [27] demonstrated the existence of an IL-12-independent pathway of IFN-*γ* induction by Gram-negative bacteria in mice in which IFN-*β* and IL-18 act synergistically. *Brucella* has induced the production of IL-18 and IFN-*β* in mice [28, 29]. Therefore, this pathway could be used to produce IFN-*γ* during *Brucella* infection in absence of IL-12. Even though IFN-*γ* can be produced independently of IL-12, we did not detect this cytokine in IL-12/*β*2-m KO spleen cells activated with live *Brucella* at one week postinfection. It is possible that IFN-*γ* increases in IL-12/*β*2-m KO after one week postinfection. Another possibility is that IFN-*γ* production by NK and other cells that are present in low numbers in spleens is underestimated when we analyzed whole splenocytes. Further, it is possible that other cell types present in other organs than spleen are responsible for residual IFN-*γ* production in IL-12/*β*2-m KO.

Macrophages are key elements in innate immune responses and recognition of *Brucella* components [30]. Herein, we investigated the involvement of IFN-*γ* and IL-12/*β*2-m in *Brucella*-induced IL-12 and TNF-*α* production by macrophages. As expected, macrophages from IL-12/*β*2-m KO mice showed no production of IL-12 when they were stimulated with live *Brucella* compared to normal synthesis of this cytokine by IFN-*γ* KO and wild-type cells. As for TNF-*α* production, no statistically significant difference was observed between KO mouse macrophages compared to C57BL/6. Since macrophages are considered the main cells of *Brucella* residence in the host, we infected these cells and tested them as targets for primed splenocytes from KO and wild-type mice in a cytotoxic assay. Pathogenesis induced by *Brucella* is the product of a complex series of interactions between the bacteria and different components of the immune system. One interaction of interest is between CD8+ CTL and *Brucella*-infected macrophages. In this study, specific lysis of infected macrophages was detected in wild-type and IFN-*γ* KO but not in IL-12/*β*2-m KO mice. IL-12/*β*2-m KO mice fail to assemble and express MHC class I molecules on the cell surface, and, therefore, these animals are devoid of functional CD8+ *αβ* T cells. Thus, the lack of functional CD8+ T cells might be the reason why we did not detect macrophage lysis by IL-12/*β*2-m KO splenocytes. Recently, Durward et al. [31] have identified two CD8+ T cell epitopes in *B. melitensis* that induced IFN-*γ* production and specific killing in vivo. Their work reinforced the important aspect of inducing *Brucella*-specific CD8+ T cells to achieve an efficient host response to this pathogen.

Collectively, we have demonstrated that IFN-*γ* and IL-12/*β*2-m are important components of host immune response to control *Brucella* infection. However, lack of endogenous IFN-*γ* is more crucial to immunity against this pathogen than lack of functional CD8+ T cells and IL-12.
